# Automatic Liver Segmentation on Volumetric CT Images Using Supervoxel-Based Graph Cuts

**DOI:** 10.1155/2016/9093721

**Published:** 2016-04-05

**Authors:** Weiwei Wu, Zhuhuang Zhou, Shuicai Wu, Yanhua Zhang

**Affiliations:** ^1^College of Electronic Information and Control Engineering, Beijing University of Technology, Beijing 100124, China; ^2^College of Life Science and Bioengineering, Beijing University of Technology, Beijing 100124, China

## Abstract

Accurate segmentation of liver from abdominal CT scans is critical for computer-assisted diagnosis and therapy. Despite many years of research, automatic liver segmentation remains a challenging task. In this paper, a novel method was proposed for automatic delineation of liver on CT volume images using supervoxel-based graph cuts. To extract the liver volume of interest (VOI), the region of abdomen was firstly determined based on maximum intensity projection (MIP) and thresholding methods. Then, the patient-specific liver VOI was extracted from the region of abdomen by using a histogram-based adaptive thresholding method and morphological operations. The supervoxels of the liver VOI were generated using the simple linear iterative clustering (SLIC) method. The foreground/background seeds for graph cuts were generated on the largest liver slice, and the graph cuts algorithm was applied to the VOI supervoxels. Thirty abdominal CT images were used to evaluate the accuracy and efficiency of the proposed algorithm. Experimental results show that the proposed method can detect the liver accurately with significant reduction of processing time, especially when dealing with diseased liver cases.

## 1. Introduction

Liver cancer is one of the most common cancers worldwide, with increasing morbidity and high mortality [1]. Computed Tomography (CT) has been widely used for clinical diagnosis of hepatic disease because of its high resolution. Accurate liver segmentation from abdominal CT scans is critical for computer-assisted diagnosis and therapy, including patient-specific liver anatomy evaluation, functional assessment, treatment planning, and image-guided surgery [2]. Traditionally, radiologists or physicians have to manually delineate the liver region slice by slice, which is tedious and time-consuming due to the large amount of data [3]. Therefore, accurate and efficient methods for liver segmentation are demanded.

Liver segmentation from CT images remains an open challenge due to the high variability in the shape and size of liver, presence of pathologies like tumor or cirrhosis, and low contrast with adjacent tissues or organs [4]. Recently, a large variety of semiautomatic and automatic methods have been developed to improve the liver segmentation procedure. These methods are commonly based on region growing [5–7], clustering [8, 9], deformable models or level sets [10–13], statistical shape models (SSMs) [14, 15], probabilistic atlases [16–18], and graph cuts [19–24]. Several comprehensive reviews of liver segmentation techniques have been conducted [25–27].

Table 1 gives an overview of selected liver segmentation methods for CT images. Semiautomatic segmentation methods tend to obtain more precise results compared to automatic methods, while automatic methods can reduce interoperator and intraoperator variability. Region growing or clustering approaches are fast and easy to implement, but they may become ineffective when the liver is inhomogeneous due to large lesions. Deformable models and level set based methods are often used as the final step to refine the segmented contour/surface to improve the accuracy, but they are sensitive to the initial contour/surface and need an iterative process which is time-consuming. Probabilistic atlases and SSMs can offer highly accurate results, but they demand sufficient training data with gold standards to generate the atlases or shape models. Graph cuts methods have been widely used in medical image segmentation because they can achieve global optimum solution. For liver segmentation, graph cuts methods based on adaptive thresholding [19], SSMs [20], probabilistic atlas [16], and deformable models [24] have been developed. However, directly constructing the graph over the voxels of CT volume data will lead to a high computational cost.

In this paper, we proposed a new method for automatic liver delineation on CT volume images using supervoxel-based graph cuts. It was highly efficient compared to other methods and provided accurate segmentation results. Both the liver volume of interest (VOI) and the foreground/background seed points for graph cuts were extracted automatically. Supervoxels of the liver VOI were generated, and the graph used for three-dimensional (3D) graph cuts segmentation was defined over the obtained VOI supervoxels.

The rest of this paper is organized as follows.  Section 2 introduces the details of the proposed method.  Section 3 describes the experiments.  Section 4 shows the experimental results, and discussion is given in Section 5. Conclusions are summarized in Section 6.

## 2. Methods

**Figure 1:**
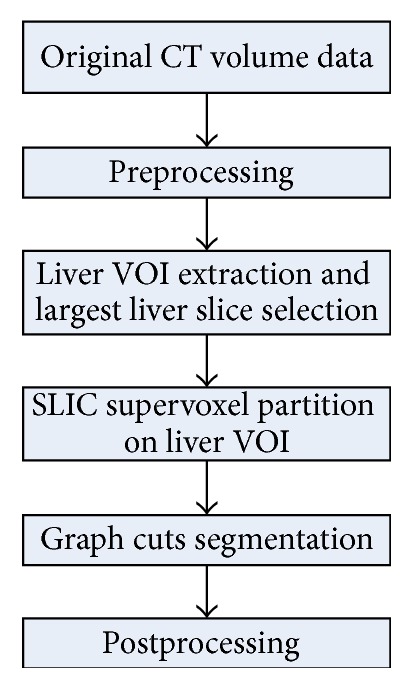
Flow chart of the proposed 3D liver segmentation method.

The flow chart of the proposed approach is illustrated in Figure 1. The proposed segmentation framework consisted of five steps. (1) In the preprocessing step, smoothing and resampling were conducted by using recursive Gaussian filtering and linear interpolation, respectively. (2) The patient-specific liver VOI extraction procedure was performed firstly by determining the region of abdomen using maximum intensity projection (MIP) [28] and thresholding methods, followed by applying a histogram-based adaptive thresholding method and morphological operations. The largest liver slice, which was the axial slice containing the maximal amount of liver tissue, was automatically selected. (3) The generation of supervoxels was performed on the liver VOI using the simple linear iterative clustering (SLIC) method [29]. (4) In the segmentation step, foreground/background seed points for graph cuts were selected on the largest liver slice, and additional background seed points on regions of heart and kidney were extracted automatically on selected heart and kidney slices. Gaussian mixture models (GMMs) were used to estimate the intensity distributions of foreground/background seeds [30]. Subsequently, the graph cuts algorithm [31, 32] was applied to the VOI supervoxels. (5) Finally, morphological opening, cavity filling, and median filtering were used to refine the segmented liver. After being resampled back to the original spatial resolution and size, the segmentation result was output for evaluation.

### 2.1. Preprocessing

The preprocessing step included smoothing and resampling. Recursive Gaussian filtering was first applied to smooth the input CT volume image *I*. Then, the smoothed CT volume image was resampled from anisotropic to isotropic voxel size (1.5 × 1.5 × 1.5 mm) by linear interpolation to obtain the preprocessed volume image *I*_pre_.

### 2.2. Liver VOI Extraction and Largest Liver Slice Selection

For computational efficiency, the region of abdomen *I*_ROA_ was firstly extracted from *I*_pre_ to remove several nonabdominal slices and voxels. Then, the liver VOI image *I*_VOI_ was determined in *I*_ROA_.

#### 2.2.1. 3D Abdominal Region Extraction

To extract the region of abdomen *I*_ROA_, a 3D abdominal bounding box (ABB) was measured using MIP and thresholding. Coronal and axial MIP images were obtained by applying the MIP algorithm to *I*_pre_. Let [*X*_min⁡_, *X*_max⁡_, *Y*_min⁡_, *Y*_max⁡_, *Z*_min⁡_, *Z*_max⁡_] be the coordinates of ABB, where *Z*_min⁡_ and Z_max⁡_ were the lower and upper bounding coordinates along *z*-axis and *X*_min⁡_, *X*_max⁡_, *Y*_min⁡_, and *Y*_max⁡_ were the in-plane bounding coordinates. The dimensions of *I*_pre_ were denoted by *u*_*x*_, *u*_*y*_, and *u*_*z*_ in *x*, *y*, and *z* directions, respectively.

**Figure 2:**
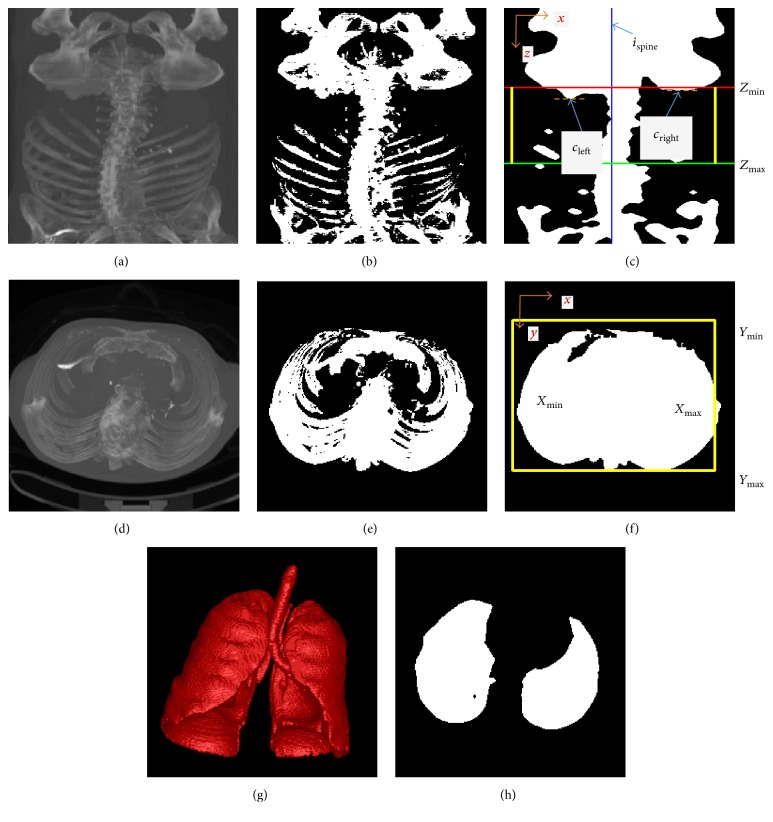
An example of the 3D abdominal region extraction. (a) Coronal MIP image. (b) The binary bone mask of (a). (c) The processed binary mask. Blue line shows the position of spine. Red and green lines show the lower and upper bounding along *z*-axis, respectively. Yellow lines are corresponding to the in-plane bounding box in (f). (d) Axial MIP image. (e) The binary abdomen mask of (d). (f) The processed binary mask. Yellow rectangle shows the in-plane bounding box. (g) The extraction of lungs. (h) The binary lung mask with the largest lung region area.

*(1) Calculation of the Lower Bounding Coordinate Z*_*min*_. For the coronal MIP image *M*_coronal_ (Figure 2(a)), which had a size of *u*_*x*_ × *u*_*z*_, segmentation of bones was performed using the Otsu algorithm [33] to obtain the binary bone mask *M*_bone_ (Figure 2(b)). Let *n*_total_ denote the total number of pixels in *M*_bone_. The number of bone pixels *n*_*b*_ (in white color) was counted.

If *n*_*b*_/*n*_total_ < 3/4, *M*_bone_ was regarded as successful bone segmentation. Otherwise, if *n*_*b*_/*n*_total_ ≥ 3/4, which meant the extraction of bones failed, Otsu algorithm with two thresholds was applied to *M*_coronal_ to generate thresholds *T*_1_ and *T*_2_ (*T*_1_ < *T*_2_); then, *M*_bone_ was obtained by applying binary thresholding [*T*_2_, *T*_max⁡_] to *M*_coronal_, where *T*_max⁡_ was the maximum CT value in Hounsfield unit (HU).

For the bone mask *M*_bone_, morphological opening with round structuring elements (radius *r* = 3), cavity filling, and median filtering were conducted to generate the processed binary bone mask *M*_bone_^*∗*^ (Figure 2(c)).

For *M*_bone_^*∗*^, the number of bone pixels was calculated in each column: *i* = 0,1,…, *u*_*x*_ − 1. Among multiple columns with the maximum number of connected bone pixels, the left-most column was regarded as the position of spine *i*_spine_ (shown as the blue line in Figure 2(c)). Then, among columns *i* = 0,1,…, *i*_spine_ − 30 and *i* = *i*_spine_ + 30,…, *u*_*x*_ − 1, the local maximums of the number of connected bone pixels, *c*_left_ and *c*_right_, were computed on both sides of the spine.

Assign *Z*_min⁡_ = min⁡(*c*_left_, *c*_right_) − 5, shown as the red line in Figure 2(c).

*(2) Calculation of the In-Plane Bounding Coordinates X*_*min*_, *X*_*max*_, *Y*_*min*_ *, and Y*_*max*_. For the axial MIP image *M*_axial_ (Figure 2(d)), Otsu algorithm with two thresholds was used to generate thresholds *T*_3_ and *T*_4_ (*T*_3_ < *T*_4_), and then binary thresholding [*T*_3_, *T*_max⁡_] was applied to *M*_axial_ to obtain the binary abdomen mask *M*_abdomen_ (Figure 2(e)).

For *M*_abdomen_, morphological closing (*r* = 1), cavity filling, and selection of the largest connected region were performed to generate the processed binary abdomen mask *M*_abdomen_^*∗*^ (Figure 2(f)).

A two-dimensional (2D) bounding box was determined based on the abdominal region (in white color) in *M*_abdomen_^*∗*^. The coordinates of the 2D bounding box were then taken as [*X*_min⁡_, *X*_max⁡_, *Y*_min⁡_, *Y*_max⁡_].

Assign [*X*_min⁡_, *X*_max⁡_, *Y*_min⁡_, *Y*_max⁡_] ← [*X*_min⁡_ − 5, *X*_max⁡_ − 5, *Y*_min⁡_ − 10, *Y*_max⁡_], as the liver was located in the right upper quadrant of the abdomen, shown as the yellow rectangle in Figure 2(f).

*(3) Calculation of the Upper Bounding Coordinate *Z_*max*_. Lungs are filled with air and have very low intensity values in CT images. By applying binary thresholding [*T*_min⁡_, −300] to *I*_pre_, where *T*_min⁡_ was the minimum CT value in HU, candidate air-filled regions were extracted. Then, the binary lung mask *I*_lung_ was obtained by selecting lung regions among the candidate air-filled regions.  Figure 2(g) shows the 3D visualization of a lung mask.

For *I*_lung_, the number of lung pixels was calculated in each axial slice *j* = 0,1,…, *u*_*z*_ − 1. The slice *j*_max⁡_ with the maximum number of lung pixels was regarded as the upper bounding of ABB (Figure 2(h)). Assign Z_max⁡_ = *j*_max⁡_, shown as the green line in Figure 2(c).

Based on the extracted 3D abdominal bounding box ABB, *I*_pre_ was cropped to obtain the region of abdomen *I*_ROA_.

#### 2.2.2. Liver VOI Extraction

In the region of abdomen, liver is the largest organ and located in the right upper quadrant of the abdomen. Also, liver is the largest object in middle axial slices of *I*_ROA_. According to this prior knowledge, the liver VOI was extracted from *I*_ROA_ by applying a histogram-based adaptive thresholding method and morphological operations.

For adaptive thresholding, a rough estimation of the liver intensity range [*T*_lower_, *T*_upper_] was calculated from the histogram of *I*_ROA_, where *T*_lower_ and *T*_upper_ denoted the minimal and maximal intensity values of liver voxels, respectively.

**Figure 3:**
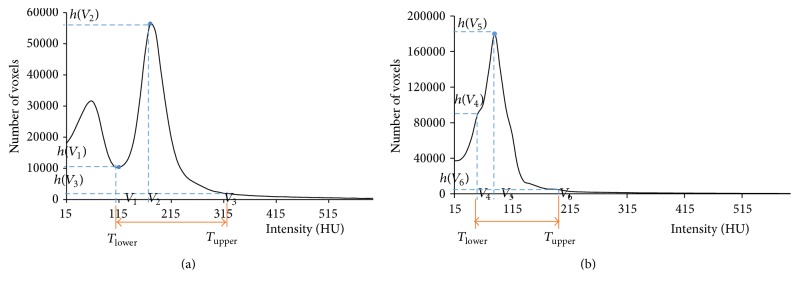
Examples of volumetric histograms in range [15, 600] HU. (a) A high contrast image with two high peaks. (b) A low contrast image with only one high peak.

By analyzing the volumetric histogram of *I*_ROA_, *I*_ROA_ could be classified as high contrast or low contrast [34]. The image with two high peaks in the histogram was regarded as high contrast (Figure 3(a)). If only one high peak was detected in the histogram, the image was regarded as low contrast (Figure 3(b)).

In Figure 3, *V* denoted the intensity value and *h*(*V*) was the histogram value corresponding to *V*. If *I*_ROA_ was a high contrast image, *V*_1_ and *V*_2_ were taken as the intensity values of the valley and the second peak, respectively; *V*_3_ was calculated by *h*(*V*_3_) = *h*(*V*_2_)/30,  *V*_3_ > *V*_2_; then, assign *T*_lower_ = *V*_1_ and *T*_upper_ = *V*_3_. If *I*_ROA_ was a low contrast image, *V*_5_ was taken as the intensity value of the peak; then, *V*_4_ and *V*_6_ were calculated by *h*(*V*_4_) = *h*(*V*_5_)/2,  *V*_4_ < *V*_5_ and *h*(*V*_6_) = *h*(*V*_5_)/30,  *V*_6_ > *V*_5_, respectively; assign *T*_lower_ = *V*_4_ and *T*_upper_ = *V*_6_.

**Figure 4:**
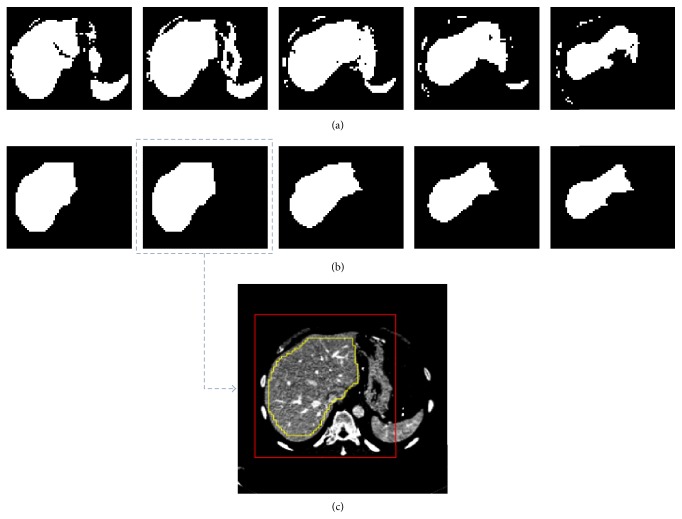
An example of liver VOI extraction and the largest liver slice selection. (a) Axial slices of the initial binary liver mask by using adaptive thresholding. (b) Axial slices of the processed binary liver mask. (c) The selected largest liver slice with the initial liver region in yellow and the liver VOI in red.

By applying binary thresholding [*T*_lower_, *T*_upper_] to *I*_ROA_, the initial binary liver mask *I*_liver_^0^ was obtained (Figure 4(a)).

For *I*_liver_^0^, selection of the largest connected component on the right side of abdomen was conducted on each axial slice to obtain the binary mask *I*_liver_^1^. Then, morphological opening (*r* = 1) and selection of the largest connected region were applied to each coronal slice of *I*_liver_^1^ to obtain the binary liver mask *I*_liver_^2^ (Figure 4(b)).

To determine the liver VOI, the bounding box of the largest 3D connected object in *I*_liver_^2^ was calculated. The liver VOI image *I*_VOI_ was taken as the obtained bounding box with a margin of ten voxels around it.

#### 2.2.3. Largest Liver Slice Selection

In the largest liver slice corresponding to the largest cross section of the liver, the liver was a whole object. Therefore, the seed points could be extracted on the largest liver slice without missing any separate parts of the liver.

To select the largest liver slice, the number of liver pixels was calculated in each axial slice of *I*_liver_^2^ (Figure 4(b)). The slice with the maximum number of liver pixels was regarded as the largest liver slice. As shown in Figure 4(c), the largest liver slice *M*_liver_ was selected with the corresponding binary liver mask *M*_liver_^*∗*^, where the red rectangle represented the liver VOI and the yellow contour denoted the liver region in *M*_liver_^*∗*^.

### 2.3. SLIC Supervoxel Partition on Liver VOI

**Figure 5:**
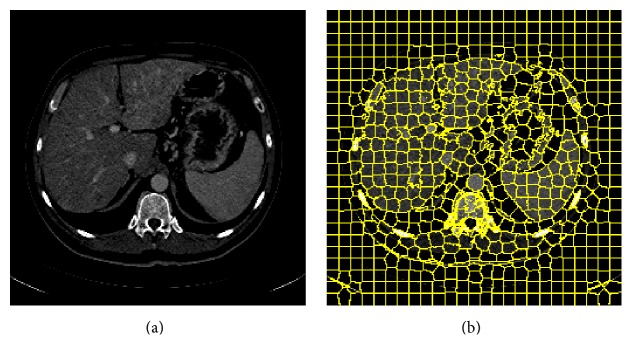
Illustration of superpixel generation. (a) Original image. (b) Supervoxels generated by the 2D simple linear iterative clustering (SLIC) algorithm. Yellow contours show the boundaries between the superpixels.

SLIC is a novel *k*-means based clustering algorithm which can generate supervoxels quickly and efficiently [29, 35]. The SLIC supervoxels have nearly uniform size, while their boundaries closely match true image boundaries.  Figure 5 shows the result of superpixel partition on one CT slice using 2D SLIC algorithm.

Let *N*_*v*_ denote the number of voxels in the liver VOI image *I*_VOI_:

(1) $$\begin{array}{c} N_{v}=N_{p}S^{3}, \end{array}$$

where *S* denoted the supervoxel spacing in each dimension and *N*_*p*_ was the desired number of supervoxels. The intensity of each supervoxel was computed as the average intensity of all voxels within the supervoxel.

### 2.4. Graph Cuts Segmentation

The liver segmentation problem can be posed as a binary labeling problem and formulated in terms of energy minimization. Graph cuts segmentation achieves an optimal solution by minimizing the energy function via the max-flow/min-cut algorithm [31, 32]. Necessary hard constraints (seed points) and intensity distributions were required for the graph cuts segmentation.

#### 2.4.1. Necessary Hard Constraints

Necessary foreground/background seed points should be selected for graph cuts. On the largest liver slice *M*_liver_, foreground seed points were sampled automatically in the liver region corresponding to the mask *M*_liver_^*∗*^. By applying morphological dilating (*r* = 20), negative operation, and binary thresholding [−50, *T*_max⁡_] to *M*_liver_^*∗*^, the background mask *M*_bkg_^*∗*^ was obtained. Then, background seed points were sampled automatically on *M*_bkg_^*∗*^ around the liver region.

Additional background seeds on regions of heart and kidney were also selected to prevent oversegmentation. In our experiments, the upper bounding of the liver VOI in *z* direction was taken as the heart slice, and one or two kidney slices were selected among slices between the lower bounding of *I*_VOI_ and the largest liver slice.

In liver CT images, regions of heart and kidney always have higher intensities compared to the liver region. Based on the histogram mentioned in Section 2.2, binary thresholding [*V*_*m*_, *T*_max⁡_] was applied to the heart and kidney slices to obtain the binary background masks *M*_heart_^*∗*^ and *M*_kidney_^*∗*^, where *V*_*m*_ denoted the intensity value of the highest peak in the histogram.

For *M*_heart_^*∗*^ and *M*_kidney_^*∗*^, morphological opening (*r* = 1) and eroding (*r* = 2) were applied. Then, the connected regions with average intensity values less than *V*_*m*_ + 10 and *V*_*m*_ + 50 were removed on *M*_heart_^*∗*^ and *M*_kidney_^*∗*^, respectively. Additional background seed points were sampled in the remaining regions of *M*_heart_^*∗*^ and *M*_kidney_^*∗*^.

In our method, the graph cuts algorithm was applied to the liver VOI supervoxels. Therefore, the seed points were all converted to the corresponding supervoxels in the liver VOI.

#### 2.4.2. Gaussian Mixture Models

For liver segmentation on CT images, there were foreground tissues including liver parenchyma, vessels, and tumors as well as background tissues including intercostal muscles, bones, heart, and kidneys. The intensity distribution of each tissue was assumed to follow Gaussian distribution [36].

Let *V*_*p*_ denote the intensity of a supervoxel *p*, and let *N* be the number of supervoxels in the foreground or background samples. The intensity distributions of foreground and background were represented as GMMs:

(2) PGMMVp=∑k=1KωkPNVp ∣ μk,σk2,PNVp ∣ μk,σk2=12πσ2exp⁡−12σ2Vp−μ2,μk=1Nk∑pVp,σk2=1Nk∑pVp2−μk2,ωk=1NNk,

where *ω*_*k*_, *μ*_*k*_, and *σ*_*k*_^2^ were the weight, mean, and variance of the *k*th Gaussian components, respectively; *K* denoted the number of Gaussian components. One GMM was used for the foreground *P*_fg_(*V*_*p*_), and another one was used for the background *P*_bkg_(*V*_*p*_). The expectation maximization (EM) algorithm [37] was applied to estimate the parameters of GMMs.

#### 2.4.3. Graph Cuts

The graph *G* = (*Q*, *W*) was created, where *Q* represented the set of nodes. The nodes comprised the liver VOI supervoxels *p*, a source terminal *Q*_*S*_, and a sink terminal *Q*_*T*_. The set of edges *W* consisted of two types of undirected edges: *n*-links (neighborhood links) connecting all unordered pairs (*p*, *q*) of neighboring supervoxels in the liver VOI and *t*-links (terminal links) connecting each supervoxel to the two terminals.

Let *L* = (*l*_1_,…, *l*_*p*_,…, *l*_*N*_*p*__) be a binary vector whose components *l*_*p*_ specified assignments to supervoxels *p*; *l*_*p*_ ∈ {0,1}, with *l*_*p*_ equaling 0 for background and 1 for foreground (i.e., liver). Vector *L* defined a segmentation, and the goal was to assign a unique label to each *p* by minimizing the following energy function *E*(*L*):

(3) $$\begin{array}{c} E\left(\;L\;\right)=\alpha \underset{p\in P}{\sum }R\left(\;l_{p}\;\right)+\beta \underset{\left(p,q\right)\in W}{\sum }B\left(\;l_{p},l_{q}\;\right), \end{array}$$

where *R*(*l*_*p*_) and *B*(*l*_*p*_, *l*_*q*_) were the regional term and boundary term, respectively; *α* and *β* were weighting factors. The minimum cost cut could be computed in polynomial time for the two terminal graph cuts.

Table 2 gives the weights of edges in *W*. We specified that *Q*_*S*_ and *Q*_*T*_ corresponded to label 0 (background) and label 1 (liver), respectively. Two sets of the seed points were defined as foreground nodes *Q*_fg_ and background nodes *Q*_bkg_, respectively.

The regional term *R*(*l*_*p*_) specified the cost of assigning a label *l*_*p*_ to *p* based on its intensity *V*_*p*_ and the intensity probabilistic model. With the foreground and background GMMs, we defined *R*(*l*_*p*_) as negative log-likelihood:

(4) Rlp=0=−log⁡PfgVp,Rlp=1=−log⁡PbkgVp.

The boundary term *B*(*l*_*p*_, *l*_*q*_) represented the penalty of the discontinuity between two adjacent supervoxels *p* and *q* [38]. The penalty became large when *p* and *q* had similar intensity values. Consider

(5) Blp,lq=δlp,lq·1Vp−Vq2+1,δlp,lq=1,if  lp≠lq,0,if  lp=lq.

### 2.5. Postprocessing

After finishing the 3D graph cuts segmentation procedure, the resulting binary liver mask *I*_seg_ was obtained. Morphological opening, cavity filling, largest region selection, and median filtering methods were applied to *I*_seg_ to remove spicules and smooth the liver surface. Then, the binary image was resampled back to the original spatial resolution and size for further evaluation.

## 3. Experiments

### 3.1. Datasets

The MICCAI Sliver07 datasets [4] used in this study contained 30 clinical contrast-enhanced (portal venous phase) abdominal CT volume images acquired using a variety of scanners, including 20 images with expert segmentations (Sliver07-train) and 10 testing images (Sliver07-test). Most datasets were pathologic, and Sliver07-test datasets involved relatively more extreme cases. The ground truth of Sliver07-test was not publicly available, and the evaluations on Sliver07-test were performed by the organizer of the MICCAI Sliver07.

For Sliver07 datasets, the number of slices, in-plane resolution, and interslice resolution varied between 64 and 394, 0.58 and 0.81 mm, and 0.7 and 5.0 mm, respectively. The segmented liver region was defined to include the entire liver tissue and all internal structures, including vessels, tumors, and cirrhosis.

### 3.2. Evaluation Measures

To quantitatively evaluate the performance of the proposed method, five different error measures were used to measure the volumetric overlap or surface distances of the segmentation result compared to the ground truth [4]. These were volumetric overlap error (VOE) [%], relative volume difference (RVD) [%], average symmetric surface distance (ASD) [mm], root mean square symmetric surface distance (RMSD) [mm], and maximum symmetric surface distance (MaxD) [mm]:

(6) VOE=1001−A∩BA∪B,RVD=100A−BB,ASD=1SA+SB∑SA∈SAdSA,SB+∑SB∈SBdSB,SA,RMSD=1SA+SB∑SA∈SAd2SA,SB+∑SB∈SBd2SB,SA,MaxD=max⁡maxSA∈SA⁡dSA,SB,maxSB∈SB⁡dSB,SA,

where *A* denoted the segmented volume produced by the proposed method; *B* was the segmented volume by experts; *S*(*A*) denoted the set of surface voxels of *A*. The shortest distance of a voxel *v* to *S*(*A*) was defined as *d*(*v*, *S*(*A*)) = min_*s*_*A*_∈*S*(*A*)_⁡‖*v* − *s*_*A*_‖, where ‖·‖ denoted the Euclidean distance. A positive value of RVD meant oversegmentation, while a negative value meant undersegmentation.

For MICCAI Sliver07 datasets, the score of 100 indicated a perfect segmentation when all the five measures were zero [4]. The manual segmentation of the average quality (VOE = 6.4%, RVD = 4.7%, ASD = 1.0 mm, RMSD = 1.8 mm, and MaxD = 19.0) was worth the score of 75. The total running time for segmentation was also recorded.

### 3.3. Implementation

To validate the proposed method, experiments were conducted on HP EliteBook 8470w (Intel Core 2.40 GHz CPU and 4 GB RAM). The proposed algorithm was implemented in C++ under Visual Studio 2008, with the use of SLIC code by Achanta et al. [29] (http://ivrl.epfl.ch/research/superpixels) and max-flow/min-cut code by Boykov and Kolmogorov [31] (http://vision.csd.uwo.ca/code/). The GMM algorithm was implemented using the open source computer vision toolkit OpenCV (http://www.opencv.org/), with seeds initialization performed by the* k*-means++ algorithm [39]. The insight segmentation and registration toolkit ITK (http://www.itk.org/) and the visualization toolkit VTK (http://www.vtk.org/) were used for basic 3D image processing and 3D visualization of segmentation results, respectively.

The key parameters of the proposed algorithm were determined experimentally on five datasets, which were selected randomly from Sliver07-train. SLIC supervoxels were generated with step *S* = 3 and compactness *m* = 20. The number of components *K* in foreground/background GMM models was set to *K* = 5. In graph cuts segmentation, the *n*-links of the graph were specified to 6-neighborhood connectivity. The weighting factors of the energy function were set to *α* = 0.01 and *β* = 100.

In the experiments, a larger value of *K* had no obvious improvement on the segmentation result. A smaller value of *K* led to imprecise segmentations in some cases, due to the presence of pathologies like tumors, similar intensity with adjacent tissues, and low CT image quality.

In the supervoxel partition step, when *S* was set to a larger step size, fewer number of supervoxels would be generated, resulting in a lower segmentation accuracy. However, as shown in Table 3, when *S* = 3, the number of the liver VOI supervoxels was suitable, on an average of 46 × 53 × 51 supervoxels. A smaller value of *S* led to more supervoxels, but it would take a longer time for supervoxel generation.

## 4. Results

**Figure 6:**
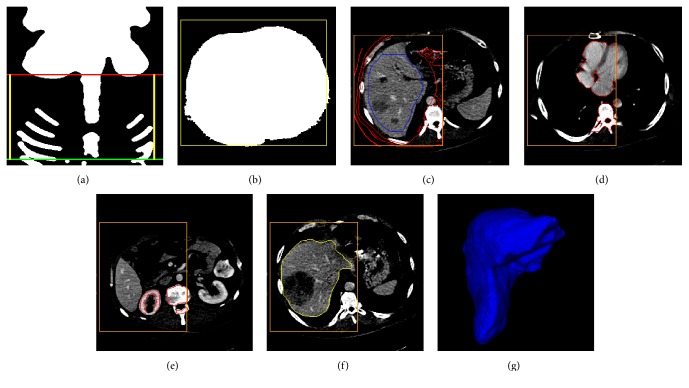
An example of liver segmentation using the proposed method. (a) and (b) show the 3D abdominal region extraction. (c) The selected largest liver slice with initial liver region in blue contour and background regions in red contours. From (c) to (f), orange rectangles show the liver VOI. (d) and (e) show the heart and kidney slices, respectively, with additional background regions in red contours. (f) The segmented result after postprocessing in yellow contour. (g) The reconstructed 3D liver volume.

Figure 6 illustrates an example of liver segmentation using the proposed method. Figures 6(a) and 6(b) show the 3D abdominal region extraction using MIP and thresholding methods. In Figures 6(c)–6(f), the orange rectangles show the extracted liver VOI, which was obtained by analyzing the histogram and by using adaptive thresholding and morphological methods.  Figure 6(c) shows the largest liver slice with initial liver region in blue and background region in red. Foreground and background seed points were sampled in the liver and background region, respectively. To avoid oversegmentations of heart and kidney, additional background seeds were extracted on the heart and kidney slices, as shown in Figures 6(e) and 6(f). The segmented liver after postprocessing is shown as the yellow contour in Figure 6(f).  Figure 6(g) shows the 3D liver volume reconstructed by using surface rendering algorithms in VTK.

**Figure 7:**
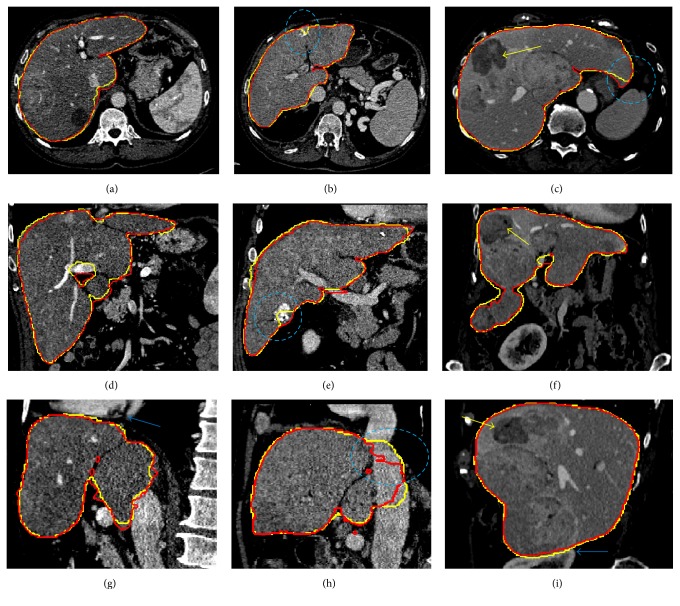
Illustrations of the segmentation results. Each column shows slices of one case in the axial, coronal, and sagittal directions, respectively. The contour of the ground truth is in red. The contour of the segmented liver by the proposed method is in yellow.

Figure 7 illustrates some representative slices of the segmentation results from three Sliver07-train datasets compared with expert segmentations, where the red contours indicate the expert segmentations and the yellow contours are the segmentation results by the proposed method. Each column shows slices of one specific case in axial, coronal, and sagittal directions, respectively. It can be seen that the livers extracted by the proposed method are comparable to the expert segmentations.

In Figures 7(g) and 7(i), our method succeeded in separating the liver from heart and kidney to avoid oversegmentations. In the third column of Figure 7, the presence of tumors in the liver also could be handled. However, there were still some small oversegmentation or undersegmentation errors occurring near the liver boundaries, mainly due to the low contrast near the liver boundaries. Undersegmentation errors occurred at the tip of the liver (Figure 7(c)) and high intensity regions near the liver edges (Figures 7(b) and 7(e)). Oversegmentation error was mainly at the place of vena cava (Figure 7(h)).

Table 3 shows examples of the reduction of volume size after each step before graph cuts segmentation. It can be seen that the extraction of liver VOI and generation of supervoxels can significantly reduce the volume size, therefore reducing computational cost.

For Sliver07-train datasets, Table 4 shows the comparative results of the proposed method with the traditional graph cuts method (TGC) [32] and the semiautomatic graph cuts method on SLIC supervoxels (SGC). SGC was similar to our method except that there was no step of liver VOI extraction and largest liver slice selection, and the extraction of seed points was conducted by manual interaction on one or two axial slices. In addition, for SGC, the original CT image was firstly converted to the 8-bit image in the intensity range [0, 255] by using linear contrast stretching with a fixed intensity window. Then, the 8-bit image was used in the procedures of supervoxel generation and GMM modeling. For Sliver07-train datasets, the proposed method had a VOE and RVD of 7.54% and 4.16% and an ASD, RMSD, and MaxD of 0.95 mm, 1.94 mm, and 18.48 mm, respectively. The total runtime was 21.21 s on average. It can be seen that, compared with TGC, our method had a great improvement on the time efficiency, which was achieved mainly by using the supervoxels. Compared with SGC, the proposed automatic segmentation method took less runtime and could obtain more robust segmentation without user interaction.

The evaluation results on the Sliver07-test datasets are presented in Table 5, comparing results of the proposed algorithm and human experts. The average VOE of 7.87%, RVD of 1.31%, ASD of 1.286 mm, RMSD of 2.498 mm, and MaxD of 23.563 mm were obtained, which were comparable to the performance of human experts. The total runtime, including the time taken for VOI extraction, largest liver slice selection, supervoxel generation, and graph cuts segmentation, was 27.04 s on average.

Table 6 shows the quantitative comparative results of the proposed method with previous methods for the Sliver07-test datasets. It can be seen that, compared with automatic segmentation methods, semiautomatic methods might have a higher accuracy. In automatic graph cuts based segmentation methods, Li [20] obtained the highest accuracy by using deformable graph cuts with SSM based initialization. The proposed method was comparable to the performance of human experts and the method of Platero [18]. Compared with other methods, the proposed method significantly improved the time efficiency of liver segmentation.

## 5. Discussion

### 5.1. Contributions of This Study

In this paper, an efficient supervoxel-based graph cuts method was proposed for automatic liver segmentation from CT images.

To reduce computational time and memory requirement, the proposed method effectively incorporated the graph cuts method with supervoxels partition. The SLIC method was applied to generate supervoxels in the liver VOI. By constructing the graph over supervoxels, the number of nodes in the graph was reduced significantly. Therefore, the computational complexity of graph cuts was decreased.

An automatic liver VOI extraction method was introduced, which contained two steps. First, the region of abdomen was extracted by using MIP and thresholding methods. By analyzing the region of bones in the coronal MIP image and finding the largest lung slice in the extracted lung region, unnecessary nonliver slices were removed. By measuring the in-plane abdominal bounding box in the axial MIP image, more nonliver voxels were excluded. Second, the liver VOI was extracted from the region of abdomen by applying adaptive thresholding and morphological methods. The adaptive threshold values were calculated by estimating the liver intensity range in the volumetric histogram. According to the prior knowledge of the liver's location and size, an initial binary liver mask was generated and the liver VOI was determined based on the binary liver mask. Voxels outside the liver VOI were removed.

For the purpose of extracting foreground and background seed points automatically, the largest liver slice and heart/kidney slices were extracted to generate foreground and background regions by using adaptive thresholding methods. On the largest liver slice, foreground seeds were sampled inside the liver region without missing any separate parts of liver. To tackle the problem of separating the liver from heart and kidney with similar intensities, additional background seeds were selected on the heart and kidney slices. Then, sufficient seed points were used to estimate the intensity distributions of foreground and background.

### 5.2. Comparison with Previous Work

Compared with the traditional graph cuts method (Table 4), the proposed approach can realize 3D liver segmentation in an automatic and fast manner. The main improvements come from (1) constructing the graph over supervoxels instead of the voxels, which can greatly reduce the number of nodes in the graph, therefore improving the efficiency of the energy minimization procedure, and (2) generating seed points automatically instead of manual interaction. The foreground and background seed points were sampled in the initial liver region and background regions, respectively. This can avoid interoperator or intraoperator variability and improve the robustness of the proposed method.

As shown in Table 6, in the semiautomatic graph cuts based methods, Chen [23] incorporated domain knowledge of the intensity, location, and spatial connectivity into the optimization framework, and Peng [24] incorporated appearance and intensity constraints for both the liver and tumor. Unlike Chen's and Peng's methods, both the liver VOI and foreground/background seed points were extracted automatically in our method. Prior knowledge of liver and histogram information were introduced such that the extraction procedures of VOI and seed points were conducted in an adaptive way.

In the automatic graph cuts methods, Platero [18] combined probabilistic atlases with graph cuts, and Li [20] incorporated SSM with graph cuts. Compared to the initialization procedure based on atlases or shape models in Platero's and Li's methods, the proposed method generated hard constraints for graph cuts segmentation based on histogram analysis and simple morphological operations. Also, the nodes of the graph corresponded to supervoxels instead of the voxels in the liver VOI. Although the segmentation accuracy of our method was lower than that of Li's SMM initialized graph cuts method [20], it was comparable to the performance of human experts. Moreover, the proposed method was faster than other graph cuts based methods. Such a fast segmentation speed may be required in practical applications like computer-assisted ablation needle trajectory (path) planning in image-guided hepatic ablation therapy [40]. Needle trajectory planning involves segmentation of all relevant structures including liver, tumor, hepatic vessels, and other surrounding organs [41, 42]. In cases like CT-guided hepatic ablation procedures, the time efficiency of needle trajectory planning is highly demanded. Therefore, it would be beneficial if the liver segmentation procedure was performed in a fast way with a low computational cost.

Compared with other previous methods based on SSMs, probabilistic atlases, and deformable models, no training process of atlases or shape models was needed for initialization in the proposed method. As shown in Figure 7, although there was a great variation in the intensity, shape, and position of liver among different datasets, the proposed algorithm correctly segmented liver from surrounding organs or tissues.

### 5.3. Limitations

Figure 9(c) shows an inadequate segmentation at the long and thin boundaries of the liver. This is mainly due to the similarity in intensity and the shrinking bias of graph cuts minimization. Surface refinement work could be applied to get a better result. Also, the energy function of graph cuts may be improved to obtain better performance on the tiny parts of liver.

**Figure 8:**
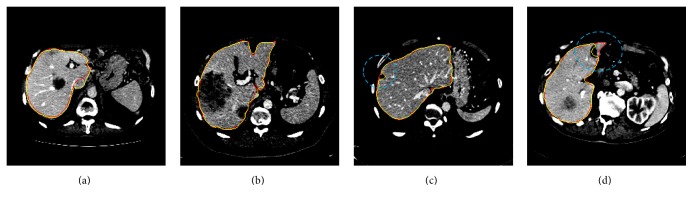
Illustrations of the segmentation results compared with expert segmentations from Sliver07-train datasets. The contour of the ground truth is in red. The contour of the segmented liver by the proposed method is in yellow.

**Figure 9:**
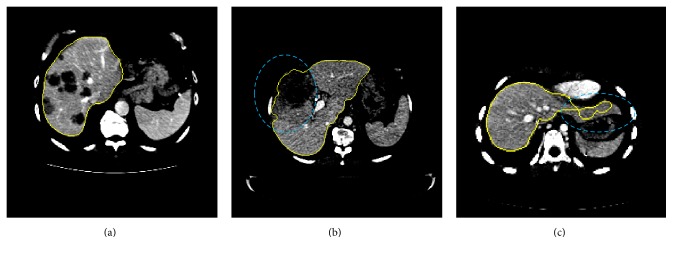
Illustrations of the segmentation results from Sliver07-test datasets. The contour of the segmentation result of the proposed method is in yellow.

On most pathological cases, our approach can handle the presence of tumors in liver, as shown in Figures 8(a), 8(b), and 9(a). However, in Figures 8(c), 8(d), and 9(b), tumors near the liver boundaries still might be misclassified. For such cases, algorithms for generating automatically additional foreground seeds on both the liver and tumor regions could be incorporated in future work.

Another limitation of this study is that the number of datasets for evaluation is small. More datasets should be used to evaluate the performance of the proposed method in the future.

## 6. Conclusions

This paper presented a new method for automatic liver CT image segmentation using SLIC supervoxels-based graph cuts. The liver VOI was extracted to reduce computational cost. Foreground and background seed points were generated automatically on the largest liver slice and on additional heart and kidney slices to avoid undersegmentations or oversegmentations. By supervoxel partition and liver VOI extraction, the computation time of the proposed method was reduced to less than one minute, while accurate segmentation result can be obtained. In the future, methods for reducing undersegmentation or oversegmentation errors need to be designed to improve the accuracy of the proposed method, especially for pathological cases with tumors. Also, more datasets for evaluation are needed.

## Figures and Tables

**Table 1:** Overview of liver segmentation methods for CT images: auto = automatic; semi = semiautomatic; VOE = volumetric overlap error; RVD = relative absolute volume difference; MaxD = maximum symmetric surface distance; DSC = dice similarity coefficient; RG = region growing; DM = deformable model; SSM = statistical shape model; PA = probabilistic atlas; GC = graph cuts; local = from local hospitals; Sliver07 = MICCAI 2007 grand challenge in segmentation of liver datasets.

Study	Year	Method	Accuracy	Dataset	Auto	Time (s)	CPU (GHz)
Kumar et al. [7]	2013	RG	DSC = 98%	local	Auto	40/slice	—

Goryawala et al. [8]	2014	Clustering + RG	DSC = 92% RVD = 2.78%	local	Semi	10.96/slice	—

Peng et al. [10]	2014	DM	VOE = 6.10% RVD = −0.00% MaxD = 16.80 mm	Sliver07	Semi	180	3.16 GHz 4 GB RAM

Kainmüller et al. [15]	2007	SSM + DM	VOE = 6.09% RVD = −2.86% MaxD = 18.69 mm	Sliver07	Auto	900	Intel 3.2 GHz

Linguraru et al. [16]	2010	PA + DM	DSC = 96.2% VOE = 2.20% ASD = 1.20 mm	Sliver07	Auto	—	—

Platero and Tobar [18]	2014	PA + GC	VOE = 7.60% RVD = −0.50% MaxD = 24.70 mm	Sliver07	Auto	261.35	Intel Xeon E5520 2.27 GHz

Massoptier and Casciaro [19]	2007	GC	DSC = 95%	local	Auto	—	—

Li et al. [20]	2015	SSM + GC	VOE = 6.24% RVD = 1.18% MaxD = 18.82 mm	Sliver07	Auto	284.95	Core(TM) i5 3.1 GHz 4 GB RAM

Chen et al. [23]	2012	GC	VOE = 4.16% RVD = 3.53% MaxD = 16.70 mm	Sliver07	Semi	60–180	Intel Core 2 2.66 GHz 3.25 RAM

**Table 2:** Graph edge weights.

Edge	Weight	For
(*p*, *q*)	*β* · *B*(*l*_*p*_, *l*_*q*_)	(*p*, *q*) ∈ *W*

(*p*, *Q*_*S*_)	0	*p* ∈ *Q*_fg_
	∞	*p* ∈ *Q*_bkg_
	*α* · *R*(*l*_*p*_ = 0)	Others

(*p*, *Q*_*T*_)	∞	*p* ∈ *Q*_fg_
	0	*p* ∈ *Q*_bkg_
	*α* · *R*(*l*_*p*_ = 1)	Others

**Table 3:** Examples of reduction of the volume size: train = Sliver07-train datasets; test = Sliver07-test datasets.

Data	Original CT [voxels]	Resampled CT [voxels]	Abdominal region [voxels]	Liver VOI [voxels]	VOI supervoxels [supervoxels]
Train number 5	512 × 512 × 319	197 × 197 × 212	163 × 175 × 200	121 × 148 × 192	40 × 49 × 64
Train number 11	512 × 512 × 388	200 × 200 × 258	172 × 136 × 148	172 × 136 × 148	57 × 45 × 49
Train number 14	512 × 512 × 129	245 × 245 × 427	217 × 162 × 138	137 × 160 × 138	45 × 53 × 46
Test number 1	512 × 512 × 502	253 × 253 × 267	216 × 219 × 122	129 × 183 × 122	43 × 61 × 40
Test number 4	512 × 512 × 165	253 × 253 × 328	234 × 202 × 176	143 × 178 × 169	47 × 59 × 56

**Table 4:** Comparative results for the Sliver07-train datasets: auto = automatic; semi = semiautomatic; TGC = traditional graph cuts method; SGC = semiautomatic graph cuts method on supervoxels.

Method	Auto	Runtime [s]	VOE [%]	RVD [%]	ASD [mm]	RMSD [mm]	MaxD [mm]	Score
TGC	Semi	180–240	11.52	6.02	1.72	3.74	31.92	57.5
SGC	Semi	30	9.73	2.00	1.64	3.26	27.82	66.1
Our method	Auto	21	7.54	4.16	0.95	1.94	18.48	75.2

**Table 5:** Quantitative evaluation of segmentation result for Sliver07-test datasets.

Data number	Runtime [s]	VOE [%]	RVD [%]	ASD [mm]	RMSD [mm]	MaxD [mm]
1	27.04	9.37	6.68	1.54	2.77	26.84
2	30.76	9.22	5.41	1.37	2.23	21.66
3	31.47	5.98	−1.68	1.14	2.02	19.73
4	36.88	7.16	2.97	1.19	2.38	18.02
5	23.93	7.60	−2.21	1.37	2.85	30.51
6	27.13	8.94	1.34	1.75	4.29	41.46
7	10.31	7.08	1.16	1.07	2.12	25.46
8	16.91	6.90	2.46	1.10	1.80	15.19
9	7.52	9.57	−1.00	1.31	2.58	22.10
10	12.03	6.89	−2.03	1.02	1.94	14.66
Average	27.04	7.87	1.31	1.29	2.50	23.56

**Table 6:** Comparative results with previous methods for the Sliver07-test datasets.

Method	Runtime [s]	Auto	VOE [%]	RVD [%]	ASD [mm]	RMSD [mm]	MaxD [mm]	Score
Peng [10]	180	Semi	6.10	−0.00	0.90	1.60	16.80	81.8
Chen [23]	60–180	Semi	4.16	3.53	0.72	1.26	16.70	81.5
Peng [24]	120–180	Semi	4.58	1.08	0.68	1.45	16.88	83.4
Li [20]	285	Auto	6.24	1.18	1.03	2.11	18.82	77.9
Platero [18]	261	Auto	7.60	−0.50	1.30	2.90	24.70	70.5
Our method	27	Auto	7.87	1.31	1.29	2.50	23.56	71.4
